# A Comprehensive Analysis of Clinical, Biochemical, and Polysomnographic Characteristics in Patients With Type 2 Diabetes Mellitus With and Without Obstructive Sleep Apnea

**DOI:** 10.7759/cureus.59734

**Published:** 2024-05-06

**Authors:** Pulkit Dhiman, Parminder Singh, Saurabh Arora, Anil Kashyap, Prannav Jain, Manavjot Singh, Jaskaran Singh, Akashdeep Singh, Fnu Suhani, Anmol Singh, Manjeet K Goyal, Ashita R Vuthaluru

**Affiliations:** 1 Gastroenterology, Dayanand Medical College and Hospital, Ludhiana, IND; 2 Endocrinology, Dayanand Medical College and Hospital, Ludhiana, IND; 3 Pulmonology, Dayanand Medical College and Hospital, Ludhiana, IND; 4 Medicine and Surgery, Dayanand Medical College and Hospital, Ludhiana, IND; 5 Internal Medicine, Dayanand Medical College and Hospital, Ludhiana, IND; 6 Pulmonary Medicine, Dayanand Medical College and Hospital, Ludhiana, IND; 7 Medicine, Dayanand Medical College and Hospital, Ludhiana, IND; 8 Internal Medicine, Global Heart & Super Speciality Hospital, Ludhiana, IND; 9 Gastroenterology and Hepatology, Dayanand Medical College and Hospital, Ludhiana, IND; 10 Anesthesia and Critical Care, Maharishi Markandeshwar Institute of Medical Sciences and Research, Ambala, IND

**Keywords:** obesity-related illnesses, waist hip, obstructive sleep apnea (osa), osa, diabetes mellitus type 2

## Abstract

Background: Obstructive sleep apnea (OSA) has been a significant contributor to mortality all across the globe. The most attributing factors to pathogenesis are metabolic syndrome, obesity, diabetes, and so on, but the indicators of its early detection are still elusive.

Objective: The study aimed to compare the clinical, biochemical, and polysomnographic characteristics of type 2 diabetes patients with and without OSA.

Design and methods: This cross-sectional study was conducted at the Department of Medicine and Endocrinology Unit of Dayanand Medical College and Hospital, Ludhiana. A total of 584 patients with type 2 diabetes were assessed using the Berlin questionnaire, with 302 fulfilling the criteria for a high risk of OSA. Out of 302 patients who met the criteria for the high-risk category, 110 patients underwent a sleep study.

Results: Three hundred and two patients satisfying the inclusion and exclusion criteria were enrolled in the study. A total of 110 patients underwent a sleep study, of which 68 (61.8%) had evidence of OSA. The waist-to-hip ratio was considerably higher in the OSA patients than in the non-OSA group (1.09 vs 0.930, p = 0.001). HbA1c >7% was found in 58.8% of OSA patients contrary to 38.1% of non-OSA patients. Fasting plasma glucose levels (>126 mg/dl) were identified in a substantially larger proportion of OSA patients than the non-OSA patients (64.7% vs 45.2%, p = 0.04). Similarly, peripheral neuropathy was found more commonly in the OSA patients than in the non-OSA patients (47% vs. 26.1%, p = 0.02). Prevalence of retinopathy, nephropathy, coronary artery disease, stroke, heart failure, and peripheral vascular disease did not differ significantly between the two groups.

Conclusions: OSA frequently occurs among individuals diagnosed with type 2 diabetes mellitus. The prompt identification of OSA within this demographic is imperative to pinpoint those at an elevated risk of succumbing to conditions such as peripheral neuropathy, the exacerbation of glycemic control, and the onset of unmanaged hypertension. Moreover, there exists a positive correlation between the waist-to-hip ratio and the prevalence of OSA in persons with type 2 diabetes mellitus, highlighting the critical role of waist-to-hip ratio assessments in this patient population.

## Introduction

Obstructive sleep apnea (OSA) represents a significant public health concern, characterized by the repetitive obstruction of the upper airway during sleep [1]. This condition, characterized by recurrent episodes of complete or partial airway collapse, results in intermittent hypoxemia and is often associated with cyclical fluctuations in heart rate, blood pressure, and autonomic nervous system activity [2]. The pathophysiological consequences of these episodes extend beyond mere sleep disruption, encompassing a spectrum of cardiovascular disorders, high blood pressure, insulin resistance, type 2 diabetes mellitus (T2DM), and increased mortality [3].

Diving into the intricate relationship between OSA and T2DM unveils a complex interplay, potentially bidirectional in nature. This multifaceted connection posits that autonomic neuropathy, a complication of diabetes, might disrupt the central regulation of respiration and the neural reflexes governing the upper respiratory tract. Such disturbances could incite or exacerbate sleep-disordered breathing patterns [4].

Emerging from the realm of epidemiology, a robust body of evidence suggests that OSA could be a significant factor in the onset of T2DM, independent of obesity and other traditionally recognized risk factors [5]. This hypothesis is underpinned by the myriad physiological stressors associated with OSA, including recurrent episodes of nocturnal hypoxia, systemic inflammation, sympathetic nervous system overactivity, sleep fragmentation, and the consequent activation of the hypothalamic-pituitary-adrenal axis [6]. These elements collectively contribute to a physiological environment conducive to the development of diabetes.

The narrative is further complicated by the findings of numerous cross-sectional and cohort studies, which highlight the alarmingly high prevalence of OSA among individuals with T2DM, transcending ethnic and demographic boundaries. While obesity and advancing age are commonly implicated as risk factors for both conditions, the emerging consensus within the scientific community points toward an association between OSA and T2DM that is independent of these factors [7].

This study embarked on a comparative analysis of clinical, biochemical, and polysomnographic characteristics among T2DM patients, delineating those with and without OSA. The objective was to unravel the nuances of their interrelation and to shed light on the pathophysiological mechanisms that may underlie this association.

## Materials and methods

Methods

Study Design

In this cross-sectional study, the objective was to methodically compare the clinical, biochemical, and polysomnographic characteristics of patients diagnosed with T2DM, with and without coexisting OSA. Additionally, the study aimed to assess the efficacy of the Berlin questionnaire as a screening tool for OSA risk among the T2DM cohort. The Berlin questionnaire, conceptualized by Netzer et al., serves as a pragmatic and accessible instrument for identifying OSA risk within the general population [8]. Structured around three pivotal categories -- snoring behavior, daytime somnolence, and fatigue, along with disease history, demographic, and anthropometric variables -- it facilitates the stratification of participants into high- or low-OSA-risk groups. A participant’s risk level is adjudged high if positive scores are recorded in two or more categories, while a score in one or none of the categories denotes low risk [9]. The questionnaire was administered by a trained interviewer, ensuring consistent and accurate data collection.

Study Participants

The study was meticulously conducted within the Department of Medicine and Endocrinology Unit at Dayanand Medical College and Hospital, a premier tertiary care institution in Punjab. Before commencement, the institutional ethics review board sanctioned the study, ensuring adherence to ethical standards.

The Berlin questionnaire was administered to 584 patients diagnosed with T2DM, identifying 302 individuals at high risk for OSA. Among these, a subset of 110 patients consented to further assessment via sleep studies utilizing the portable RESMED APNOEA LINK device. The apnea-hypopnea index (AHI) served as the diagnostic criterion, categorizing OSA severity into mild, moderate, and severe based on specific AHI thresholds. Concurrently, the latest HbA1c results were compiled for all participants.

Diabetes mellitus patients aged between 35 and 70 years were included in the study. Patients with a history of chronic obstructive airway disease (emphysema and chronic bronchitis) and patients with structural disease of the respiratory tract (polyposis and major deformities) were excluded from the study.

Detailed evaluation regarding the presence of microvascular complications of T2DM was carried out at the time of presentation. The diagnosis of retinopathy was established by the presence of characteristic findings on fundus examination after dilating the pupils. The typical findings included retinal hemorrhages, microaneurysm, cotton wool spots, hard exudates, venous beading, intraretinal microvascular abnormality, areas of neovascularization, vitreous hemorrhage, or laser coagulation scars in at least one eye. Diabetic nephropathy was diagnosed clinically by persistently high spot urinary albumin-to-creatinine ratio ≥30 mg/g taken on the first-morning sample and/or sustained reduction in estimated glomerular filtration rate (eGFR) below 60 ml/min per 1.73 m^2^. Diabetic peripheral neuropathy was defined by symptoms of neuropathy (feeling of numbness, pain, paresthesia, or decreased sensation in the distal lower limb) along with the absence of the Achilles tendon reflex. Study subjects were considered hypertensive in case of systolic blood pressure more than 140 mm Hg, diastolic blood pressure more than 90 mm Hg, or both, or if the subject had received antihypertensive drugs. Dyslipidemia was diagnosed if the study subject had serum total cholesterol concentration of more than 420 mg/dl, triglyceride concentration of 150 mg/dl, high-density lipoprotein cholesterol concentration 40 mg/dl, or if the subjects were already being managed with antihyperlipidemic medications. Coronary artery disease and cerebrovascular accident were diagnosed based on the history given by the patient and review of the medical records.

Waist circumference measurement. For women, the waist circumference is precisely recorded at the narrowest segment between the chest and hips, while for men, it is measured at the umbilicus level. This measurement is performed with the subject in a standing posture, using a flexible tape measure, and is noted down to the nearest centimeter.

Hip circumference measurement. The hip circumference is captured at the peak circumference around the buttocks, specifically at the level of the greater trochanters. This measurement, recorded in centimeters, is taken from behind the participant.

Waist-to-hip ratio (WHR) determination. The WHR is computed by dividing the waist circumference by the hip circumference. According to WHO recommendations, the thresholds for waist circumference are set at 85 cm for men and 80 cm for women. Additionally, the WHR cutoff values are 0.90 for men and 0.80 for women, with values above these indicating a heightened risk of various health issues.

Statistical Analysis

Statistical analysis was performed using SPSS version 20 (IBM SPSS Statistics for Windows, version 20.0. Armonk, NY: IBM Corp.). Descriptive statistics are presented in percentages and mean ± standard deviation. The Mann-Whitney U-test was used for comparing continuous variables and the chi-square test was performed for comparison between categorical variables. Binary logistic regression analysis was performed to determine the association of selected variables with the severity of OSA. A p-value <0.05 was considered to be statistically significant.

## Results

In this study, a cohort of 582 individuals with diabetes was subjected to screening via the Berlin questionnaire, revealing that 302 participants (51.7%) were classified at a high risk for OSA. From this high-risk group, 110 individuals (36.4%) consented to participate in a sleep study. The findings from these sleep studies indicated that 68 of the participants (61.8%) exhibited evidence of OSA. Within this subset, the distribution of OSA severity was as follows: 66.2% of the patients were diagnosed with mild OSA, 17.6% with moderate OSA, and 16.2% with severe OSA.

The demographic analysis revealed that the mean age of individuals in the OSA group was 55.2 years, with a standard deviation of 9.2 years, while the mean age of those in the non-OSA group was 53.5 years, with a standard deviation of 8.6 years. The average duration of diabetes among the participants subjected to polysomnography was 6.4 years, with a standard deviation of 2.4 years. The male-to-female ratio among these participants was 1.44. Furthermore, the mean body mass index (BMI) was recorded at 28.4 kg/m², with a standard deviation of 4.9 kg/m², and the mean waist circumference was noted as 103.9 cm, with a standard deviation of 12.8 cm.

Hypertension was identified in 72 participants (65.4%) from the sleep study cohort, with 24 individuals (21.8%) exhibiting uncontrolled blood pressure. Detailed baseline characteristics of all participants undergoing polysomnography are systematically presented in Table 1.

**Table 1:** Baseline clinical characteristics of 110 study participants

Variable	N (%)
Age (years, mean ± SD)	54.8 ± 9.0
Male-to-female ratio	1.62
Body mass index (kg/m^2^, mean ± SD)	29.07 ± 4.9
Duration of diabetes (years, mean ± SD)	6.4 ± 2.4
Use of oral hypoglycemic agents (%)	90 (81.8)
Insulin therapy (%)	15 (13.6)
Glycosylated hemoglobin (mean ± SD)	8.9 ± 2.7
Hypertension (%)	72 (65.4)
Dyslipidemia (%)	68 (61.8)
Alcohol consumption (%)	52 (47.2)
Smoking (%)	18 (16.3)
Stroke (%)	3 (2.7)
Coronary artery disease (%)	10 (9.0)
Heart failure (%)	6 (5.4)
Peripheral vascular disease	2 (1.8)
Diabetic retinopathy (%)	40 (36.3)
Diabetic nephropathy (%)	13 (11.8)
Diabetic neuropathy (%)	43 (39.1)
Hypothyroidism (%)	14 (12.7)
Waist circumference (cm, mean ± SD)	103.9 ± 11.2
Neck circumference (cm, mean ± SD)	40.3 ± 6.1
Waist-to-hip ratio	1.004

Patients with and without OSA did not differ significantly by age, gender, BMI, duration of diabetes, insulin use, smoking, and alcohol consumption. The mean BMI in the OSA group was 29.8 kg/m^2^ and approximately one-half of the patients (45.6%) in this group had a BMI of less than 25 kg/m^2^. The mean waist circumference of patients with OSA was more as compared to patients without OSA but this could not reach statistical significance (105.1 cm vs 102.0 cm, p = 0.26). Waist circumference was significantly higher in females as compared to males in the OSA group (108.1 vs 103.2, p = 0.04). The neck circumference of patients was almost similar in both groups (40.58 cm vs 40.02 cm, p = 0.33). The WHR was significantly higher in patients diagnosed with OSA in comparison to the non-OSA group (1.09000 vs 930, p = 0.001). Mean average breaths/min were significantly higher in patients who didn’t have OSA as compared to patients who had OSA (19.46 vs 15.51, p = 0.04). There was no significant difference in average O_2_ saturation, lowest O_2_ saturation, and pulse rate in both groups (p > 0.05).

Hypertension was present in 73.5% of patients in the OSA group whereas 52.4% of patients in the non-OSA group had evidence of hypertension (p = 0.03). Uncontrolled blood pressure (systolic blood pressure > 140 mm/Hg or diastolic blood pressure > 90 mm/Hg or both) readings were more frequently observed in the OSA group as compared to the non-OSA group (27.9% vs 11.9%, p = 0.04). Glycosylated hemoglobin of more than 7% (>53 mmol/mol) was observed in 58.8% in the OSA group while 38.1% of patients in the non-OSA group had their glycosylated hemoglobin above 7% (p = 0.03). Fasting plasma glucose of more than 126 mg/dl was found in a significantly higher proportion of patients in the OSA group as compared to the non-OSA group (64.7% vs 45.2%, p = 0.04). Prevalence of coronary artery disease, stroke, heart failure, and peripheral vascular disease did not differ significantly between the two groups. Patients in the OSA group did not have a significantly higher prevalence of nephropathy and retinopathy as compared to the non-OSA group. Peripheral neuropathy was detected more frequently in patients with OSA as compared to the non-OSA group (47% vs 26.1%, p = 0.02). Hypothyroidism was present in 14.7% of patients with OSA whereas 9.5% of patients had hypothyroidism in the non-OSA group and the difference between the groups was insignificant (Table 2).

**Table 2:** Comparison of clinical and biochemical characteristics of type 2 diabetes patients with and without OSA OSA, obstructive sleep apnea.

Variable	Type 2 diabetes with OSA (n = 68)	Type 2 diabetes without OSA (n = 42)	p-Value
Age (years, mean ± SD)	55.2 ± 9.2	53.5 ± 8.6	0.35
Body mass index (kg/m^2^, mean ± SD)	29.8 ± 1.2	29.5 ± 1.6	0.43
Waist circumference (cm, mean)	105.1	102	0.26
Neck circumference (cm, mean)	40.58	40.03	0.33
Waist-hip ratio	1.09	0.93	0.001
Diagnosis of hypertension (%)	73.5	52.4	0.03
Uncontrolled blood pressure (%)	27.9	11.9	0.04
Macrovascular complications (%)	20.5	16.6	0.61
Retinopathy (%)	38.2	33.3	0.60
Nephropathy (%)	13.2	9.5	0.55
Neuropathy (%)	47	26.1	0.02
Fasting plasma glucose (%)	64.7	45.2	0.04
Glycosylated hemoglobin (%)	58.8	38.1	0.03

## Discussion

OSA has become a significant health concern worldwide, particularly in patients with T2DM, in whom it is linked with deterioration in glycemic parameters. Our study results show that using the Berlin questionnaire, 302 (51.7%) were found to be at high risk for OSA. Previous studies also reported similar findings. In a study conducted by Cass et al. using the Berlin questionnaire, 48.6% of patients with T2DM were categorized as having a high risk for OSA [10]. In another survey conducted in the UK using a self-administered Berlin questionnaire, 56.2% prevalence of OSA was reported in patients with T2DM [11]. Umoh et al. using the Berlin questionnaire in type 2 diabetic patients classified 49.5% of patients as high risk for OSA [12]. Numerous studies suggest higher odds of developing OSA in patients with diabetes as compared to the general population [5,13].

Out of 110 patients categorized as high risk for OSA using the Berlin questionnaire, 68 (61.8%) patients have some degree of OSA confirmed in the sleep study. Almost two-thirds of patients (66.2%) had mild OSA (AHI 5-15), whereas 17.6% and 16.2% were diagnosed with moderate (AHI 16-30) and severe OSA (AHI > 30), respectively. Several studies have reported a more severe form of OSA in patients with T2DM as compared to patients without diabetes [14]. Almost one-third of patients in our study had evidence of moderate to severe OSA, which is consistent with the previous studies. In a study conducted by Butt et al., approximately one-third of all patients with OSA had evidence of severe OSA, which is in agreement with our observation [14]. The mechanisms responsible for the more severe presentation of OSA in diabetes are not clearly understood. The postulated mechanisms include increased levels of inflammatory cytokines (IL-6 and TNF alpha), insulin resistance, and leptin resistance leading to impairment in ventilator drive, and dysautonomia [15].

The measures of fat distribution that have been shown to better predict the risk of OSA include body mass index, neck circumference, abdominal circumference, neck height ratio, and WHR. However, the observations from various studies on the relationship between these parameters and the risk of OSA are divergent. In our study, anthropometric parameters like neck circumference, abdominal circumference, and BMI were not significantly associated with the development of OSA. Interestingly, higher WHR was significantly associated with an increased likelihood of OSA. WHR emerged as the single most important anthropometric index associated with the development of OSA. Our results are in agreement with the findings of another study, which showed that waist-hip circumference was the best predictor for OSA [16]. In contrast to our study, Hang et al. showed that waist circumference and BMI were associated with an increased risk of OSA [17]. Although BMI is often used to define obesity, it does not provide precise information regarding body fat distribution [18]. These findings underscore the importance of measuring WHR for OSA risk assessment. Another interesting observation was the relatively high prevalence of patients with a BMI below 25 (45.6%) in the OSA group. This observation suggests that factors other than obesity are involved in the development of OSA in patients with T2DM.

Our study demonstrated an association between hypertension and OSA risk (p = 0.03). Additionally, uncontrolled blood pressure was more frequently encountered in patients with OSA as compared to those without OSA (27.9% vs 11.9%, p = 0.04). Numerous observational studies have described a significant association between hypertension and OSA [19]. Patients with OSA are at increased risk of having hypertension. A Spanish study conducted by Marin et al. including 1889 individuals over a 12-year period observed an increased rate of new hypertension among OSA patients compared with those without OSA, and this association remained statistically significant even after controlling for confounding factors like age and obesity [20]. In another study, uncontrolled blood pressure in blacks significantly increased the likelihood of OSA twofold [21]. The current study revealed a significant association between OSA and glycemic control as assessed by fasting plasma glucose and glycosylated hemoglobin. In contrast to our findings, another study including 279 patients with diabetes found no significant associations between OSA and glycemic control, as measured by glycosylated hemoglobin [22]. However, full polysomnography could not be performed in 78% of the patients. Our findings are in line with the observations of Fendri et al. and Aronsohn et al., who reported that untreated OSA adversely affected glycemic control [23,24]. Overall, there is sufficient data to suggest that the presence of undiagnosed and untreated OSA may be related to poor glycemic control and high blood pressure recordings in patients with T2DM. These results may have significant clinical implications in the treatment of patients with T2DM as they support the assumption that effective management of OSA may lead to improvement in glycemic status and blood pressure control.

The prevalence of coronary artery disease, heart failure, stroke, peripheral vascular disease, diabetic retinopathy, and nephropathy did not differ significantly between the patients with and without OSA. However, peripheral neuropathy was detected in a significantly higher proportion of diabetes patients in the OSA group as compared to those without OSA (47% vs 26.1%, p = 0.02). A recent study by Tahrani et al. reported a four-fold increase in the prevalence of diabetic neuropathy in diabetic patients with OSA as compared to those without OSA [25]. A meta-analysis conducted by Fujihara et al. reported an increased prevalence of OSA in patients with diabetes and diabetic neuropathy [26]. Another study revealed a significant association between diabetic autonomic neuropathy and OSA [27]. The possible explanation for increased risk or worsening of OSA in patients with diabetic neuropathy includes disturbance in sleep caused by painful neuropathy and increased collapsibility of upper airways secondary to neuropathy. Moreover, there are studies that have reported OSA as a risk factor for the development of neuropathy in diabetes [28].

However, our study had certain limitations. First, this was a cross-sectional study, and therefore causal relationship between diabetic peripheral neuropathy and OSA could not be established. Secondly, a sleep study was not performed in Berlin in low-risk-category patients, making it tough to calculate the sensitivity and specificity of the Berlin score in our population. Thirdly, tests for autonomic neuropathy could not be performed in our study.

## Conclusions

OSA has become a major health concern around the world, particularly in individuals suffering from T2DM, where it has been associated with a deterioration in glycemic indices. The WHR appears to be a more reliable marker of OSA in patients with T2DM in comparison to BMI, waist circumference, and neck circumference. The presence of OSA in T2DM patients is linked to poor glycemic control and uncontrolled hypertension. OSA in individuals with T2DM is positively correlated with the development of peripheral neuropathy raising the possibility that OSA could be an independent risk factor for the development of diabetic peripheral neuropathy. The higher prevalence of OSA in individuals with normal BMI in our study underscores the importance of screening for OSA in T2DM irrespective of BMI. Early detection of OSA in patients with T2DM and screening for metabolic abnormalities in those with OSA may improve patients' quality of life.
